# Amelioration of Ulcerative Colitis in BALB/c Mice by Probiotic-Fermented *Aegle marmelos* Juice

**DOI:** 10.1155/ijfo/5288406

**Published:** 2025-04-02

**Authors:** Pritika Sharma, Aakriti Garg, Nidhi, Vasudha Sharma

**Affiliations:** ^1^Department of Food Technology, School of Interdisciplinary Sciences and Technology, Jamia Hamdard, New Delhi, India; ^2^Department of Pharmacology, School of Pharmaceutical Education and Research, Jamia Hamdard, New Delhi, India; ^3^Centre for Translational and Clinical Research, School of Chemical and Life Sciences, Jamia Hamdard, New Delhi, India

**Keywords:** *Aegle marmelos*, *Aegle marmelos* fruit, DSS-induced animal model, functional foods, probiotic-fermented juice, ulcerative colitis

## Abstract

*Aegle marmelos* has been used traditionally in folk medicine for the treatment of gastrointestinal (GI) disorders. Fermentation using probiotics is well established to positively modulate the GI system. This study investigated the therapeutic potential of probiotic-fermented *Aegle marmelos* juice for ulcerative colitis (UC), using a mouse model. UC was induced in mice with dextran sulphate sodium (DSS), leading to weight loss, increased disease activity, and lowered antioxidant defenses. In contrast, mice treated with probiotic-fermented *Aegle marmelos* juice showed improved body weight, reduced disease activity index, and mitigated colon damage. Inflammatory biomarkers were decreased, while antioxidant activity increased. High-performance liquid chromatography analysis of the fresh and probiotic-fermented *Aegle marmelos* juice revealed an increase in potential bioactive compounds compared to its unfermented counterpart. These findings suggest that probiotic-fermented *Aegle marmelos* juice could be a promising therapeutic option for UC, countering inflammation and displaying antioxidant properties.

## 1. Introduction

Popular in Southeast Asia, the bael fruit (*Aegle marmelos*), also known as wood apple, has long been used therapeutically. As reported in the literature, it contains a high amount of phytochemicals, including alkaloids, flavonoids, and tannins, which exhibit anti-inflammatory, antioxidant, and antimicrobial effects. Prebiotic fibres in *A. marmelos* fruit have also been reported to encourage the development of good gut microbiota [1]. The bael fruit (*A. marmelos*) has been reported in the Indian indigenous systems of medicine, such as Ayurveda and Unani, to exert positive effects on gastrointestinal disorders [2, 3]. According to Dutta et al. [4], luvangetin reduces the development of ulcers by lowering oxidative stress in the gastroduodenal mucosa. Tumor necrosis factor (TNF), a proinflammatory cytokine, is reduced by the anti-inflammatory characteristics of marmelosin, a substance derived from *A. marmelos* fruit [5]. Umbelliferone, *β*-d-galactopyranoside, a coumarin present in *A. marmelos* helps in increasing the levels of glutathione peroxidase (GPx), superoxide dismutase (SOD), and catalase activity (CAT), indicating reduced oxidative stress and inflammation [6].

Probiotics and, particularly, probiotic-fermented foods have been explored for their improved health effects on the body. Due to the possible health benefits of fermented foods, awareness has grown over the past few years. Microorganisms, such as lactic acid bacteria, transform sugars, carbohydrates, and phenolics into other substances during the fermentation process, such as organic acids, alcohols, and antioxidant compounds [7]. The health of the intestines may be improved by these substances' potential anti-inflammatory and antioxidant effects. To enable consumers to benefit from bioactive characteristics, the compounds should be bioaccessible. The biotransformation of phenolic compounds during fermentation by lactic acid bacteria enhances their absorption and biological activity [7]. Phytochemicals in *A. marmelos* fruit may become more bioavailable, with metabolites potentially offering stronger anti-inflammatory and gastrointestinal health-promoting effects after fermentation [8].

Ulcerative colitis (UC) and Crohn's disease are the two primary types of inflammatory bowel disease (IBD) [9]. UC is a chronic disorder characterized by inflammation, relapses, and remissions, affecting individuals throughout their lives. In contrast with Crohn's disease, which has uneven inflammation that can affect any part of the digestive system, UC has continuous, superficial inflammation that only affects the mucosal layer and is only present in the colon and rectum [10]. A state of chronic dysregulated immune function in the mucosa is brought on by the interaction between exogenous (the makeup of regular intestinal microbiota) and endogenous factors of the host (the role of the intestinal epithelial cell barrier, innate immune system, and adaptive immune system), which are further modified by environmental factors [11].

UC is a crippling illness with several implications. It is a significantly recognized risk factor in the development of colon cancer. The primary goals in treating UC are to lessen symptoms and enhance the quality of life for the patient rather than to cure them [12].

The TNF-*α* and interleukin (IL)-6 levels are known to increase in patients with UC. According to Olsen et al. [13], the TNF-mRNA level in UC patients was considerably higher compared to that of healthy controls, particularly in those with moderate to severe disease. TNF-mRNA levels in UC patients rose in direct proportion to the Ulcerative Colitis Disease Activity Index (UCDAI) score. In a study by Li et al. [14], it was observed that compared to both patients with inactive UC and controls, patients with active UC had noticeably more IL-6.

The disease has no known cure, and some patients may not benefit from treatment or suffer negative side effects. Sulfasalazine and corticosteroids are two anti-inflammatory medications mostly used for the management of UC [15]. These medications reduce inflammation but have a number of side effects; resultantly, UC patients often have an impaired quality of life as a result of the ongoing condition [16]. Therefore, research into alternative UC treatments is necessary.

Thus, the purpose of this research was to ascertain whether fermented *A. marmelos* juice could be beneficial in dextran sulphate sodium (DSS)–induced UC in BALB/c mice. The primary aim of this study was to evaluate the therapeutic potential of fermented *A. marmelos* juice in treating UC, using a DSS-induced mouse model. The objectives of this study are (1) to investigate whether fermentation enhances the anti-inflammatory properties of *A. marmelos* phytochemicals, (2) to assess the efficacy of fermented *A. marmelos* juice in reducing colonic inflammation in BALB/c mice, and (3) to evaluate the potential therapeutic applications of fermented *A. marmelos* juice for UC treatment.

## 2. Material and Methods

### 2.1. Ethical Statement

Since the study does not need any protected or endangered species, special permission was not needed for the collection of the fruits, and Jamia Hamdard (deemed to be university) gave permission for the collection of the fruits (*A. marmelos*) for the study. The protocol for the animal experiment was authorised by the Institutional Animal Ethics Committee (IAEC) of Jamia Hamdard (Registration Number 173/GO/Re/S/2000/CPCSEA), New Delhi, India (IAEC Proposal No. 1584).

### 2.2. Chemicals

Reference standards for marmelosin, umbelliferone, and luvangetin were bought from Sigma-Aldrich. Probiotic lactic acid bacteria (*Lactobacillus plantarum* MTCC 2941) were obtained from the Microbial Type Culture Collection Centre, Chandigarh. *Lactiplantibacillus plantarum* strain is a whole-genome sequenced bacterium that produces lactic acid. It is aerobic and requires 48 h of incubation for growth. It is equivalent to ATCC8014, CCM1904, and DSM20205 (where ATCC is the American Type Culture Collection, CCM is the Centre for Culture Collection of Microorganisms, and DSM is the German Collection of Microorganisms and Cell Cultures). It is known to enhance intestinal barrier function and has antioxidant functions according to the culture collection centers. ELISA kits were purchased from ELK Biotechnology.

### 2.3. Method of Fruit Collection and Processing

*A. marmelos* Correa, as identified by a university botanist, was obtained from the herbal garden of Jamia Hamdard (deemed to be university) campus in New Delhi, India. Processing of *A. marmelos* for pulp and juice extraction was done by washing the *A. marmelos* fruits followed by breaking their hard rind, scooping the pulp, and adding sterilized water (*w*/*w* equal amounts) to it. Then, the seeds and fibres were removed using a 20-mesh-size stainless steel sieve, followed by thermal/pasteurization treatment (80°C for 10 min) and storage (4°C–6°C).

### 2.4. Fermentation of *A. marmelos* Juice Medium

Fifty milliliters of the pasteurized juice (80°C for 10 min) was placed in each 250 mL conical flask during the fermentation tests. All samples were inoculated with a 24-h-old *L. plantarum* (5% *v*/*v*; 6 log CFU/mL) culture and incubated at 34°C for 72 h and agitation speed 108 rpm, resulting in final probiotic counts of 9 log CFU/mL. The basis for the parameters was a previously conducted study for optimization of the fermentation process using the Box–Behnken design. The fermentation temperature, level of inoculum, and agitation speed were taken as input variables in order to maximize the responses of bacterial concentration, antioxidant activity, and polyphenols. Thereafter, it was stored at a temperature of 4°C.

### 2.5. High-Performance Liquid Chromatography (HPLC) Analysis of Bioactives in *A. marmelos*

HPLC analysis was done to quantitatively evaluate the chemical components (marmelosin, umbelliferone, and luvangetin) in fresh, pasteurized, and probiotic-fermented *A. marmelos* juice, respectively. Accurately weighed, 5 mg each of marmelosin, umbelliferone, and luvangetin reference standards (Sigma-Aldrich) were diluted in 25 mL of HPLC grade methanol to create 200 g/mL stock solutions. Serial dilutions in HPLC grade methanol were used to prepare working solutions with concentrations of 1, 2, and 4 *μ*g/mL. To obtain an appropriate concentration range of marmelosin, umbelliferone, and luvangetin, the stock solutions were quantitatively transferred to 10 mL volumetric flasks. For the analysis of these compounds, a 20 *μ*L loop Rheodyne injector and HPLC connected to a photodiode array (PDA) detector were employed. The mobile phase included methanol and water at a flow rate of 1.0 mL/min, and the stationary phase was made up of a reverse-phase Bondapak TM C-18 column (300 3.9 mm id, 125 Å, 10 *μ*m film thickness). At 254 nm, the detector's wavelength was set. All samples were filtered via a nylon membrane filter (Millipore, 13 mm diameter, 0.45 mm thickness) before analysis using a glass syringe-mounted filter holder. The peak area was plotted against reference standard concentrations to create calibration curves [17].

### 2.6. Experimental Animals

BALB/c mice (male, 20–25 g in weight) were procured from the animal house facility of Jamia Hamdard, New Delhi, India. Animals were retained under normal conditions of temperature (23°C ± 1°C), relative humidity (55% ± 10%), and a cycle of 12 h/12 h light/dark and were given a standard diet of pellets and water (ad libitum) for 7 days before the initiation of the study. They were kept in cages made with polypropylene with a wire mesh top at the animal house at Jamia Hamdard.

### 2.7. Induction of Colitis

Colitis was induced by the procedure explained by Kasinathan et al. [18]. Briefly, UC was induced in experimental animals by administering 2% DSS in drinking water (w/v) from 0 to 14 days. After induction of UC (7 days of administering DSS), animals were randomly divided into five groups (*n* = 8 in each group), namely, Group 1: healthy control (received distilled water); Group 2: disease control (received 2% DSS in drinking water); Group 3: standard control (received 100 mg/mL sulfasalazine, po); Group 4: *A. marmelos* juice–treated group (1 mL, po); and Group 5: fermented *A. marmelos* juice–treated group (1 mL, po). The treatment was initiated on Day 7 and continued till Day 14 through an intragastric route. After the completion of the treatment duration, a blood sample was collected (postsedation with chloroform) using the retro-orbital route and then sacrificed by cervical dislocation [19].

### 2.8. Disease Activity Index (DAI)

During the procedure, once a day, loss in weight, rectal bleeding, and stool consistency symptoms were observed. The following criteria were used to score the DAI [20]:

− 0: no loss of weight, regular bowel movements, and no rectal bleeding;
− 1: weight loss of 1%–5%, normal stool consistency, and no rectal bleeding;
− 2: weight loss of 5%–10%, loose stools, and no rectal bleeding;
− 3: weight loss of 10%–20%, normal stool consistency, and gross rectal hemorrhage; and
− 4: weight loss of more than 20% and diarrhea.

### 2.9. Biochemical Parameter Assessment

After 14 days of the experiment, blood and colon tissue samples were collected from each group to determine the serum TNF-*α*, SOD, and IL-6 levels. Blood sample collection and evaluation were done according to the procedure mentioned in the manual provided by the manufacturer. Samples were collected and placed in a serum separator tube. Clotting was done for 2 h at room temperature, followed by centrifugation for 20 min at 1000 rpm. Colon tissue samples were stored in aliquots at −20°C. Prior to homogenization, tissues were weighed and thoroughly rinsed in precooled phosphate buffer saline (PBS) to eliminate any remaining blood. The tissues were cut into small pieces and homogenized in fresh lysis buffer using a glass homogenizer on ice (microtissue grinders). PBS (*w* : *v* = 1 : 9) was used as the lysis buffer for all tissues. An ultrasonic cell disrupter was used to sonicate the resultant suspension until the solution became clear. Following a 5-min, 10,000 rpm centrifugation of the homogenates, the supernatant was collected and kept at temperatures less than or equal to −20°C for storage. The level was determined using ELISA kits (ELK Biotechnology).

### 2.10. Statistical Analysis

The data are shown as mean ± standard deviation after each ELISA analysis, which was carried out in triplicate. Tukey's test was employed after a one-way analysis of variance (ANOVA) to compare the variations between the groups. Statistics were considered significant at *p* values < 0.05. GraphPad Prism 9.2.0 software was used to conduct this analysis.

## 3. Results and Discussions

### 3.1. Analysis of Bioactives in *A. marmelos*

Analysis in the current work was carried out for the quantification of marmelosin, umbelliferone, and luvangetin from *A. marmelos* Correa. (*A. marmelos*) because of the common occurrence and medicinal value of these substances.

The results show three bioactive compounds measured across fresh, pasteurized, and fermented juice samples. The fresh juice contained 2.80 ± 0.04*  μ*g/mL marmelosin, which decreased slightly to 2.50 ± 0.25*  μ*g/mL after pasteurization and remained similar at 2.52 ± 0.14*  μ*g/mL in the fermented sample. Umbelliferone showed the highest concentration among the three compounds in fresh juice at 3.76 ± 0.05*  μ*g/mL. The levels decreased to 2.85 ± 0.02*  μ*g/mL after pasteurization but showed a slight recovery to 3.14 ± 0.09*  μ*g/mL in the fermented sample. Fresh juice contained 2.98 ± 0.05*  μ*g/mL luvangetin, which decreased to 2.20 ± 0.01*  μ*g/mL after pasteurization. The fermented sample showed intermediate levels at 2.63 ± 0.02*  μ*g/mL. Fresh juice generally contained the highest levels of all three bioactive compounds (Table 1).

Table 1 represents the amount of bioactives present in different formulations, namely, fresh juice, pasteurized juice, and fermented juice of *A. marmelos* fruit. The concentration of bioactives was highest in fresh juice.

Pasteurization appeared to reduce the concentrations of all compounds. Fermentation seemed to partially restore some of the compounds' levels compared to pasteurization, though not to fresh juice levels. The presence of statistical markers (a, b, c, d, f) suggests there are significant differences between some of these values. The pattern suggests that both processing methods (pasteurization and fermentation) affect the bioactive compound content, with pasteurization showing the most pronounced reduction in concentrations. This could be important for understanding how processing methods impact the potential health benefits of *A. marmelos* juice.

The presence of marmelosin, umbelliferone, and luvangetin in *A. marmelos* aligns with previous phytochemical studies. Sharma et al. [21] reportedly characterized these compounds as key bioactive constituents in *A. marmelos*, linking them to various therapeutic properties. Our study suggested that the presence of marmelosin, umbelliferone, and luvangetin was comparable in the fermented, fresh, and pasteurized juice of *A. marmelos* fruit. Our results are in accordance with the studies undertaken by Shinde et al. [22] and Lanjhiyana et al. [23], wherein marmelosin and umbelliferone were found in the range of 2–10 *μ*g mL^−1^. Victoria et al. [24] separated different compounds in the ethanol extract of *A. marmelos* by HPLC using chloroform as the mobile phase and revealed the presence of 13 bioactive components with values ranging from 2.2 to 3.4 *μ*g mL^−1^. The values were 2.12 *μ*g mL^−1^ for marmelosin and 2.30 *μ*g mL^−1^ for umbelliferone.

The observed reduction in bioactive compounds after pasteurization is consistent with thermal degradation patterns documented in similar fruit products. The relatively stable levels of marmelosin across processing (2.80–2.50 *μ*g/mL) might be explained by its structural stability. The partial recovery of compounds during fermentation might be explained by microbial biotransformation. Recent work by Sidhu and Zafar [8] suggested that certain fermentation processes can enhance the bioavailability of phenolic compounds through enzymatic release from bound forms. These findings have practical implications for product development.

### 3.2. Effect of Fermented *A. marmelos* Juice on Body Weight and DAI

In a mouse model of DSS-induced colitis, the effects of fermented juice from *A. marmelos* were assessed in the current study. Compared to other animal models, this one has a number of benefits. For instance, the clinical signs and symptoms of the acute phase of DSS-induced colitis, including weight loss, diarrhea, occult blood in stools, and frank rectal bleeding, are similar to those of human colitis [25].

The UC symptoms developed within 5 days of administering 2% DSS in mice without mortality. Weight loss, stool consistency, and rectal bleeding symptoms were observed once a day in each experimental animal during the experiment. Kasinathan et al. [18] reported the development of colitis on the 4th day by the same procedure. They noted continuous diarrhea, weight loss, and blood in stools in experimental mice similar to the results of our study.

Two percent DSS administration in the disease control group significantly (*p* < 0.05) decreased the weight (15%) of experimental animals compared with the healthy control group. We observed a significant (*p* < 0.05) increase in body weight (11%) after 7 days of intragastric administration of fermented *A. marmelos* juice compared to the DSS-induced UC group (8%).

DSS administration caused significant weight loss (15%) in the disease control group, which is a typical symptom of DSS-induced colitis. This weight loss is consistent with the established understanding that DSS causes inflammatory damage to the intestinal epithelium, leading to reduced food intake and impaired nutrient absorption. Treatment with fermented *A. marmelos* juice showed a protective effect, leading to an 11% weight gain compared to the untreated DSS group. This suggests that the juice may help ameliorate the inflammatory effects of DSS and support recovery of intestinal function. This finding aligns with previous research on *A. marmelos*'s anti-inflammatory properties. A study by Behera et al. [20] demonstrated similar protective effects of *A. marmelos* against experimental colitis, showing improvements in body weight. The weight recovery seen here is particularly significant because body weight is considered a reliable indicator of disease severity and recovery in DSS colitis models. The 11% improvement suggests meaningful therapeutic potential for fermented *A. marmelos* juice in managing UC symptoms.

The DAI scores of different groups are represented in Table 2. The healthy control group showing a DAI of 0 establishes the baseline of normal health with no inflammatory symptoms. The disease control group's DAI of 3 indicates severe UC symptoms induced by DSS, characterized by significant weight loss (10%–20%), normal stool consistency, and gross rectal hemorrhage. The treatment outcomes show a hierarchical improvement. Fermented *A. marmelos* juice reduced DAI to 2, indicating moderate improvement. Both the developed nutraceutical and sulfasalazine achieved a DAI of 1, showing substantial improvement. The statistical significance (*p* < 0.0001) between disease control and treatment groups confirms the therapeutic effectiveness of all interventions. The findings align with previous research by Behera et al. [20], which assessed the DAI and found that the groups of rats that were treated with various doses of *A. marmelos* fruit extract showed significant improvement as compared to the IBD control group.

DAI: 0: no loss of weight, regular bowel movements, and no rectal bleeding;

1: weight loss of 1%–5%, normal stool consistency, and no rectal bleeding;

2: weight loss of 5%–10%, loose stools, and no rectal bleeding;

3: weight loss of 10%–20%, normal stool consistency, and gross rectal hemorrhage; and

4: weight loss of more than 20% and diarrhea.

### 3.3. Effect of Various Treatments on Biochemical Parameters of Mice Having DSS-Induced UC

Results of various treatments on BALB/c mice having DSS-induced UC are provided in Table 3.

The majority of studies on UC indicate that probiotics suppress nuclear factor kappa-B (NF-*κ*B) mechanisms. They modify key cell signaling pathways involved in inflammation by reducing TNF-*α* production, which decreases IkB degradation and phosphorylation, particularly of IkB [26, 27].

According to Castillo et al. [28], giving healthy mice probiotics increased TNF-*α* synthesis and secretion at the locations that trigger the gut immune response. After the infection, the probiotic treatment was continued to protect the host by reducing the production of TNF-*α* and boosting the production of IL-6 in the lamina propria of the small intestine, which primarily affects the immunological effector site of the gut.

After 14 days of the experiment, TNF-*α* in tissue was found to significantly (*p* < 0.05) increase in the disease group compared with the healthy control group, whereas the level of TNF-*α* significantly (*p* < 0.05) decreased within the group treated with the developed nutraceutical as compared to the disease control group, showing the efficacy of the developed nutraceutical. Similarly, the level of TNF-*α* in serum after 14 days of treatment significantly (*p* < 0.0001) declined in the nutraceutical-treated group as compared to the disease control group (Table 4).

Additionally, the level of IL-6 increased significantly (*p* < 0.01) in the disease group as compared to the control group. Treatment after the developed nutraceutical decreased the level of IL-6 in tissue as well as serum as compared to the disease control group; however, the levels were not significant (Table 3). Overall, we observed that the developed nutraceutical decreased inflammatory markers in serum as well as in tissue, showing its potential anti-inflammatory activity and efficacy for the remission of UC. In alignment with our study, Kasinathan et al. [18] reported an increase in IL-6 and TNF-*α* in DSS-induced colitis in mice, which was decreased after treatment with *A. marmelos* extract.

Furthermore, the level of SOD decreased significantly in tissue (*p* < 0.0001) as well as in serum (*p* < 0.01) after 14 days of administration with DSS compared to the healthy group. Seven-day treatment with nutraceuticals significantly (*p* < 0.05) increased the levels of SOD in tissue as well as serum compared to the disease control group, showing anti-inflammatory activity of the developed nutraceutical. SOD is an antioxidant enzyme that neutralizes free radicals, hence showing protective action. An increase in the level of SOD after administration of the developed nutraceutical suggests increased activity of the innate antioxidant enzyme. Following an erythrocyte catastrophe caused by a disease, SODs are the predominant intracellular antioxidants that are present or released into the bloodstream; their plasma/serum activity differs by several orders of magnitude from equivalent erythrocyte levels [29, 30]. Therefore, the developed nutraceutical protects the colon from damage caused by DSS through its potent antioxidant properties.

## 4. Conclusions

The results of this study highlight the promising therapeutic potential of probiotic-fermented *A. marmelos* juice in the management of UC. The findings demonstrate that treatment with this fermented juice leads to significant improvements in body weight, reduced disease activity, and mitigation of colon damage in a mouse model of UC induced by DSS. Moreover, the treatment was associated with a decrease in inflammatory biomarkers and an enhancement in antioxidant activity. HPLC analysis revealed that fermentation increased the concentration of bioactive compounds in *A. marmelos* juice, which may contribute to its therapeutic effects. These results suggest that probiotic-fermented *A. marmelos* juice could serve as an effective and natural therapeutic strategy for UC, offering anti-inflammatory and antioxidant benefits. For further extensive studies, the gut microbiota of the mice could be analysed. Long-term effects of the fermented *A. marmelos* juice can also be studied. In addition, the mechanism of action of the juice could also be explored. Further research is warranted to explore its efficacy in human subjects and to understand the underlying mechanisms in greater detail.

## Figures and Tables

**Table 1:** Bioactive components identified from different types of study samples through HPLC analysis.

**Type of *Aegle marmelos* juice**	**Marmelosin (*μ*g/mL)**	**Umbelliferone (*μ*g/mL)**	**Luvangetin (*μ*g/mL)**
Fresh	2.80 ± 0.04	3.76 ± 0.05	2.98 ± 0.05
Pasteurized	2.50 ± 0.25^a^	2.85 ± 0.02^c^	2.20 ± 0.01^a,e^
Fermented	2.52 ± 0.14^ab^	3.14 ± 0.09^cd^	2.63 ± 0.02^af^

*Note:* All data are reported as mean ± SD (*n* = 3).

^a^ *p* < 0.001 versus fresh marmelosin.

^b^ *p* < 0.001 versus pasteurized marmelosin.

^c^ *p* < 0.0001 versus fresh umbelliferone.

^d^ *p* < 0.001 versus pasteurized umbelliferone.

^e^ *p* < 0.0001 versus fresh luvangetin.

^f^ *p* < 0.0001 versus pasteurized luvangetin.

**Table 2:** Disease activity index of different animal groups.

**Groups**	**Disease activity index**	**No. of animals**
Healthy control	0	8
Disease control	3^a^	8
DSS+*Aegle marmelos* juice	2^abc^	8
DSS+developed nutraceutical	1^b^	8
DSS+sulfasalazine	1^b^	8

^a^ *p* < 0.0001 versus healthy control.

^b^ *p* < 0.0001 versus disease control.

^c^ *p* < 0.01 versus disease control.

**Table 3:** Tabulation of various treatments on tissue samples of BALB/c mice having DSS-induced ulcerative colitis.

**Groups**	**TNF-*α* of tissue (pg/mL)**	**SOD of tissue (ng/mL)**	**IL-6 of tissue (pg/mL)**
Healthy control	44.16 ± 3.69	4.42 ± 0.49	9.11 ± 2.08
Disease control	54.34 ± 3.46^a^	0.42 ± 0.42^b^	17.57 ± 2.28^c^
DSS+*Aegle marmelos* juice	43.30 ± 2.94^d^	0.93 ± 0.59^e^	16.16 ± 1.53^a^
DSS+developed nutraceutical	45.34 ± 2.58	1.60 ± 0.66^e^	13.59 ± 2.14
DSS+sulfasalazine	44.15 ± 4.04^d^	1.92 ± 0.56^af^	12.48 ± 1.60

*Note:* All data are reported as mean ± SD (*n* = 3). Tukey's test is used after a one-way analysis of variance to determine whether two values are significantly different (*p* 0.05).

*^a^ p* < 0.05 versus healthy control.

^b^ *p* < 0.0001 versus healthy control.

^c^ *p* < 0.01 versus healthy control.

^d^ *p* < 0.05 versus disease control.

^e^ *p* < 0.001 versus disease control.

^f^ *p* < 0.01 versus disease control.

**Table 4:** Tabulation of various treatments on serum samples of BALB/c mice having DSS-induced ulcerative colitis.

**Groups**	**TNF-*α* of serum (pg/mL)**	**SOD of serum (ng/mL)**	**IL-6 of serum (pg/mL)**
Healthy control	5.82 ± 1.10	2.83 ± 0.48	8.64 ± 1.87
Disease control	33.42 ± 1.87^a^	0.16 ± 0.02^b^	13.73 ± 2.09
DSS+*Aegle marmelos* juice	35.86 ± 2.89^a^	1.12 ± 0.17^d^	10.94 ± 2.82
DSS+developed nutraceutical	10.04 ± 2.35^cd^	1.26 ± 1.02^d^	9.48 ± 1.78
DSS+sulfasalazine	31.66 ± 0.27^ac^	0.93 ± 0.58^d^	9.20 ± 1.43

*Note:* All data are reported as mean ± SD (*n* = 3). Tukey's test is used after a one-way analysis of variance to determine whether two values are significantly different (*p* < 0.05).

^a^ *p* < 0.0001 versus healthy control.

^b^ *p* < 0.01 versus healthy control.

^c^ *p* < 0.0001 versus sulfasalazine group.

^d^ *p* < 0.0001 versus sulfasalazine group.

## Data Availability

The data that support the findings of this study are available on request from the corresponding author. The data are not publicly available due to privacy or ethical restrictions.
